# Whole genome sequencing of the monomorphic pathogen *Mycobacterium bovis* reveals local differentiation of cattle clinical isolates

**DOI:** 10.1186/s12864-017-4249-6

**Published:** 2018-01-02

**Authors:** Moira Lasserre, Pablo Fresia, Gonzalo Greif, Gregorio Iraola, Miguel Castro-Ramos, Arturo Juambeltz, Álvaro Nuñez, Hugo Naya, Carlos Robello, Luisa Berná

**Affiliations:** 1 Unidad de Biología Molecular, Institut Pasteur de Montevideo, Montevideo, Uruguay; 2 Unidad de Bioinformática, Institut Pasteur de Montevideo, Montevideo, Uruguay; 3 Departamento de Bacteriología, División de Laboratorios Veterinarios (DI.LA.VE.) “Miguel C. Rubino”, Montevideo, Uruguay; 4 Departamento de Bioquímica, Facultad de Medicina, Universidad de la República, Montevideo, Uruguay

**Keywords:** Bovine tuberculosis, Comparative genomics, Phylogenetics, Genetically monomorphic bacteria, European 1

## Abstract

**Background:**

Bovine tuberculosis (bTB) poses serious risks to animal welfare and economy, as well as to public health as a zoonosis. Its etiological agent, *Mycobacterium bovis*, belongs to the *Mycobacterium tuberculosis* complex (MTBC), a group of genetically monomorphic organisms featured by a remarkably high overall nucleotide identity (99.9%). Indeed, this characteristic is of major concern for correct typing and determination of strain-specific traits based on sequence diversity. Due to its historical economic dependence on cattle production, Uruguay is deeply affected by the prevailing incidence of *Mycobacterium bovis.* With the world’s highest number of cattle per human, and its intensive cattle production, Uruguay represents a particularly suited setting to evaluate genomic variability among isolates, and the diversity traits associated to this pathogen.

**Results:**

We compared 186 genomes from MTBC strains isolated worldwide, and found a highly structured population in *M. bovis.* The analysis of 23 new *M. bovis* genomes, belonging to strains isolated in Uruguay evidenced three groups present in the country. Despite presenting an expected highly conserved genomic structure and sequence, these strains segregate into a clustered manner within the worldwide phylogeny. Analysis of the non-pe/ppe differential areas against a reference genome defined four main sources of variability, namely: regions of difference (RD), variable genes, duplications and novel genes. RDs and variant analysis segregated the strains into clusters that are concordant with their spoligotype identities. Due to its high homoplasy rate, spoligotyping failed to reflect the true genomic diversity among worldwide representative strains, however, it remains a good indicator for closely related populations.

**Conclusions:**

This study introduces a comprehensive population structure analysis of worldwide *M. bovis isolates*. The incorporation and analysis of 23 novel Uruguayan *M. bovis* genomes, sheds light onto the genomic diversity of this pathogen*,* evidencing the existence of greater genetic variability among strains than previously contemplated.

**Electronic supplementary material:**

The online version of this article (10.1186/s12864-017-4249-6) contains supplementary material, which is available to authorized users.

## Background

Bovine tuberculosis (bTB) is a chronic respiratory disease of livestock characterized by the development of granulomas in affected tissues, caused by *Mycobacterium bovis*. bTB has serious animal welfare and economic consequences, affecting animal performance and the trade value of sub-products [1]. Despite milk pasteurization and cattle sanitary programs that initially succeeded to control bTB in many developed countries, wildlife reservoirs contribute to disease spillover back to domesticated animals, hampering control efforts [1]. *M. bovis* is also a zoonotic pathogen, being of major concern in the developing world, where human populations are at greater risk for transmission due to close animal-human interactions, and high HIV prevalence puts a greater number of immunocompromised individuals at risk [1, 2]. The absence of active surveillance programs and limited epidemiological studies in many countries have underestimated the local prevalence and its impact on humans locally, as well as worldwide [1, 3].

*M. bovis* belongs to the *Mycobacterium tuberculosis* complex (MTBC), a group of closely related species that share 99.9% of their nucleotide sequences, and have identical 16S rRNA genes [4, 5]. While originally considered to be a genetically monomorphic group, current evidence points to the existence of considerable genomic diversity among strains [6–8]. It has been shown that the population structure of *M. bovis* is the result of a series of clonal expansions where clones acquired high frequencies within the population [9]. To date, four clonal complexes of *M. bovis* have been defined based on distinct spoligotype signatures and deletions; European 1, European 2, African 1, African 2. These distinct spoligotypes derived from the ancestral BCG-like spoligotype SB0120 [10–15]. Nonetheless, standardized epidemiological methods for strain typing within the MTBC, such as spoligotyping, exhibits a high propensity for homoplasy [16]. On the other hand, single nucleotide polymorphisms (SNPs) are well distributed throughout the genome in both intragenic and intergenic regions, and have low reverse mutation rates and homoplasy indexes [16]. Therefore, SNPs arise as a reliable and robust tool for establishing phylogenetic relationships and for population structure studies of the MTBC [17].

Uruguay has the world’s highest number of cattle per capita (3.6), with over 12 million bovines. Although bTB prevalence has been low for the past 50 years due to the implementation of a national surveillance program [18], several outbreaks were reported between 2011 and 2013. Importantly, the country’s economy is largely dependent on the cattle industry [19]. Costly control programs and significant production decreases caused by *M. bovis*, mainly affecting the dairy cattle industry, appreciably threaten the country’s economy.

In the present study, we set out to characterize the genomic variability among *M. bovis* isolates present in the country. We sequenced and analyzed the genomic relationship of 23 *M. bovis* Uruguayan isolates, obtained from representative dairy farms in Uruguay, to worldwide strains selected to represent the highest clonal complex diversity available. Subsequent comparative genomics allowed us to explore their genomic variability and to determine local diversity. To assess the relevance of spoligotyping as a complimentary source of information on the variability of strains, we analyzed the isolates´ spoligotype patterns in silico. We found that, despite presenting the expected highly conserved genomic structure, these strains displayed key variability traits that contributed to the formation of a distinctly structured population.

## Results

### Whole genome sequencing, assembly and genotyping of 23 Uruguayan strains

The calculated coverage, N50 and genome sizes for the 23 sequenced local strains range from 21X to 161X, 44,571 to 107,175 bp and 3.49 to 4.50 Mbp, respectively. Detailed information on the sequencing statistics can be found in the Additional File 1: Figure S1. Epidemiological data for the selected Uruguayan strains of *M. bovis* is shown in Additional file 2: Table S1. All 23 strains were found to have 1 rRNA operon. Strain MbURU-002 exhibits the highest number of predicted CDS, which correlates to it having the largest genome size among the strains. The total numbers of tRNA, ranges from 50 to 58 genes. Strain-specific detailed information can be found in in Additional file 3: Figure S2. In silico spoligotyping of the sequenced Uruguayan strains showed five different patterns: SB0274 (35%), SB0145 (30%), SB0130 (26%), SB0140 (4%), and SB1072 (4%).

### Phylogenomics of *M. bovis* strains portray structured populations

**Fig. 1:**
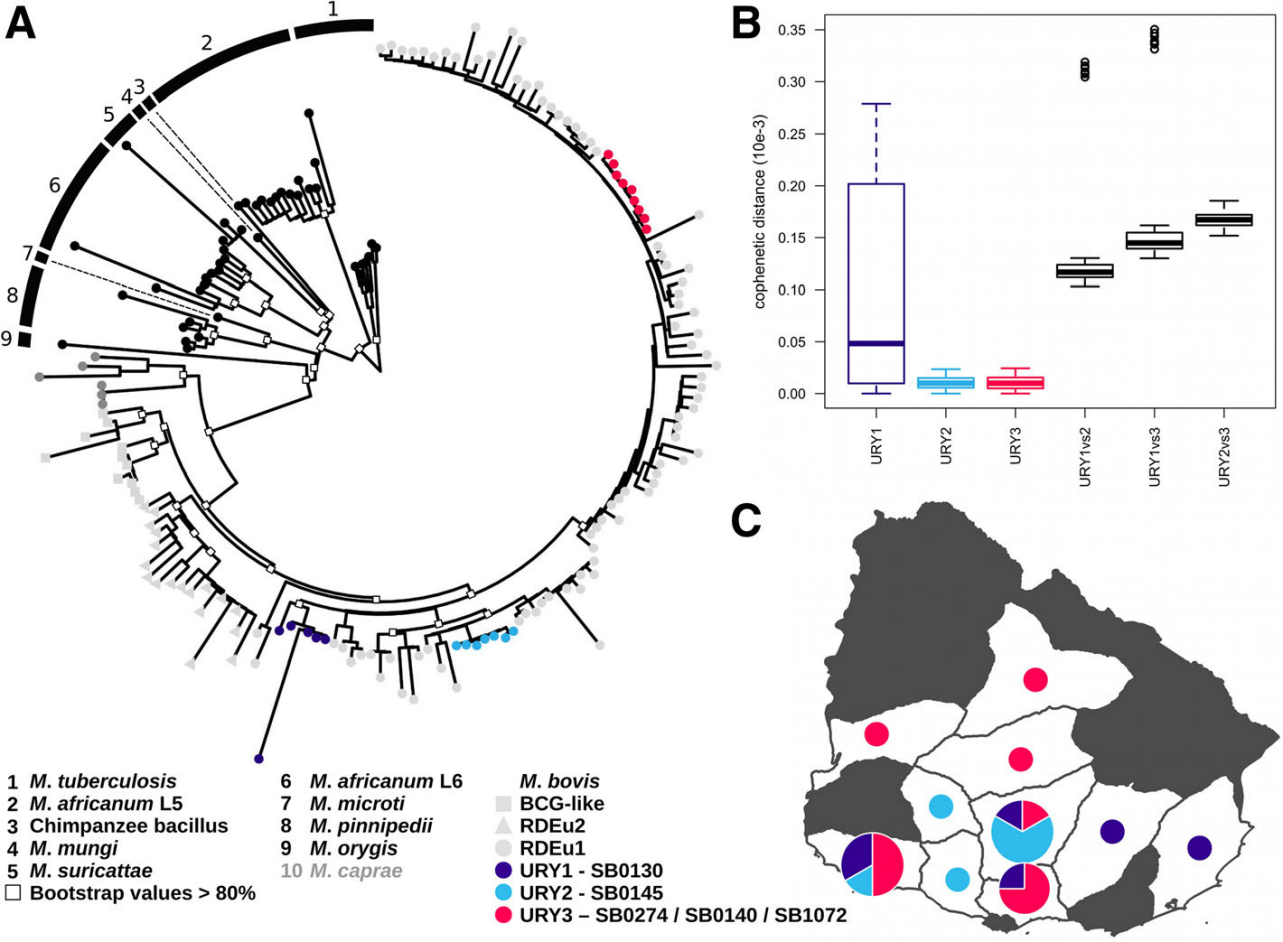
(**a**) Maximum likelihood phylogeny of *Mycobacterium tuberculosis* complex obtained based on 2370 core genes, (**b**) Cophenetic distance, showing the diversity, within the three Uruguayan groups (URY1, URY2, URY3) and between them, (**c**) Distribution by group of the 23 *M. bovis* isolates in Uruguay. While *M. bovis* was isolated from all but four of the departments of this country (data not shown), white areas specify those where the 23 sequenced strains where isolated from. The size of the circles is representative of the number of strains isolated from each department

**Fig. 2:**
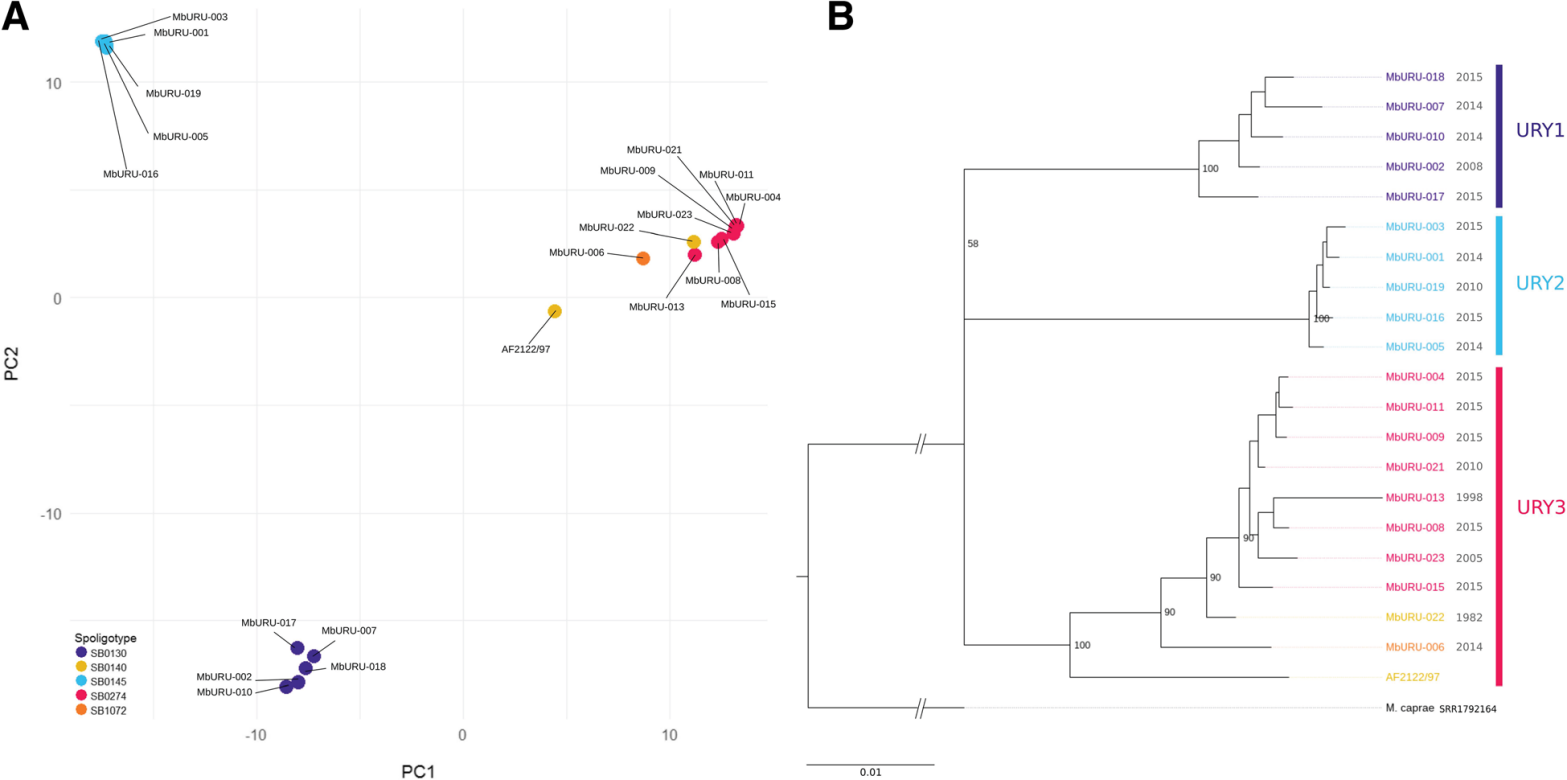
Clustering of the Uruguayan strains, evidenced by (**a**) PCA of their variants against the reference strain AF2122/97, and (**b**) phylogenetic tree of these variants obtained with RAxML. Year of isolation is showed next to each of the strain names. *Mycobacterium caprae* (SRR1792164) was chosen as outgroup

To comprehend the largest possible genetic diversity in the analysis, we included genomes from isolates with geographically distinct origins, representing three clonal complexes. We uncovered that an average of ~58% of genes are shared for any individual genome; 2370 genes are shared between the Uruguayan strains sequenced and 163 MTBC strains isolated worldwide (see Additional File 4: Table S2 for details). The phylogenetic relationships reconstructed by maximum likelihood based on the core genome (i.e. the 2370 shared genes) show three main clusters of *M. bovis*, each corresponding to the clonal complexes European 1 (Eu1), European 2 (Eu2) and BCG-like (Fig. 1a). All the Uruguayan strains cluster within the widely distributed and highly structured clonal complex Eu1. Within Eu1, the Uruguayan strains form three divergent groups (URY1, URY2, and URY3). Fig. 1c shows the geographical distribution of *M. bovis* groups URY1, URY2 and URY3 within Uruguay. URY1 shows the highest within group genetic distance (Fig. 1b), and is a well-supported cluster (bootstrap >80). URY2 shows a low within group genetic distance (Fig. 1b), and is clustered with strains from Mexico and the USA. URY3 also shows a low within group genetic distance (Fig. 1b), but groups with the most diverse and widely distributed cluster which includes strains from Argentina, Brazil, Canada, Mexico, South Korea, UK and USA (Additional File 4: Table S2). Using the core genome, we estimated π and θW for each of the groups, which measure genetic diversity both for synonymous and non-synonymous sites (Table 1). As expected, the overall variability is low. On average, URY3 is the most variable group for synonymous sites, but variability estimates based on non-synonymous sites are similar for the three groups. Variant based phylogenetic tree and Principal Component Analysis (PCA) cluster the Uruguayan strains according to their group identities (Fig. 2). Interestingly, we found there is a correlation between variants and spoligotypes. However, this is only clearly evident at the country scale, whereby MbURU-022 (SB0140) shares more variants with the rest of URY3 than with AF2122/97 (SB0140)**.**

**Table 1:** The mean number of segregating sites and estimates of synonymous and non-synonymous genetic diversity for each group. SS: Segregating sites across the core genes. π: The average pairwise differences per site. θW: Watterson’s estimator of genetic diversity based on the number of segregating sites

	Synonymous			Non-Synonymous		
	SS	π	θW	SS	π	θW
URY1	312	0.000006	0.00005	264	0.00004	0.00004
URY2	162	0.0000004	0.00002	139	0.00002	0.00001
URY3	523	0.00001	0.00005	370	0.00003	0.00004

### Variability throughout *M. bovis* genomes

**Fig. 3:**
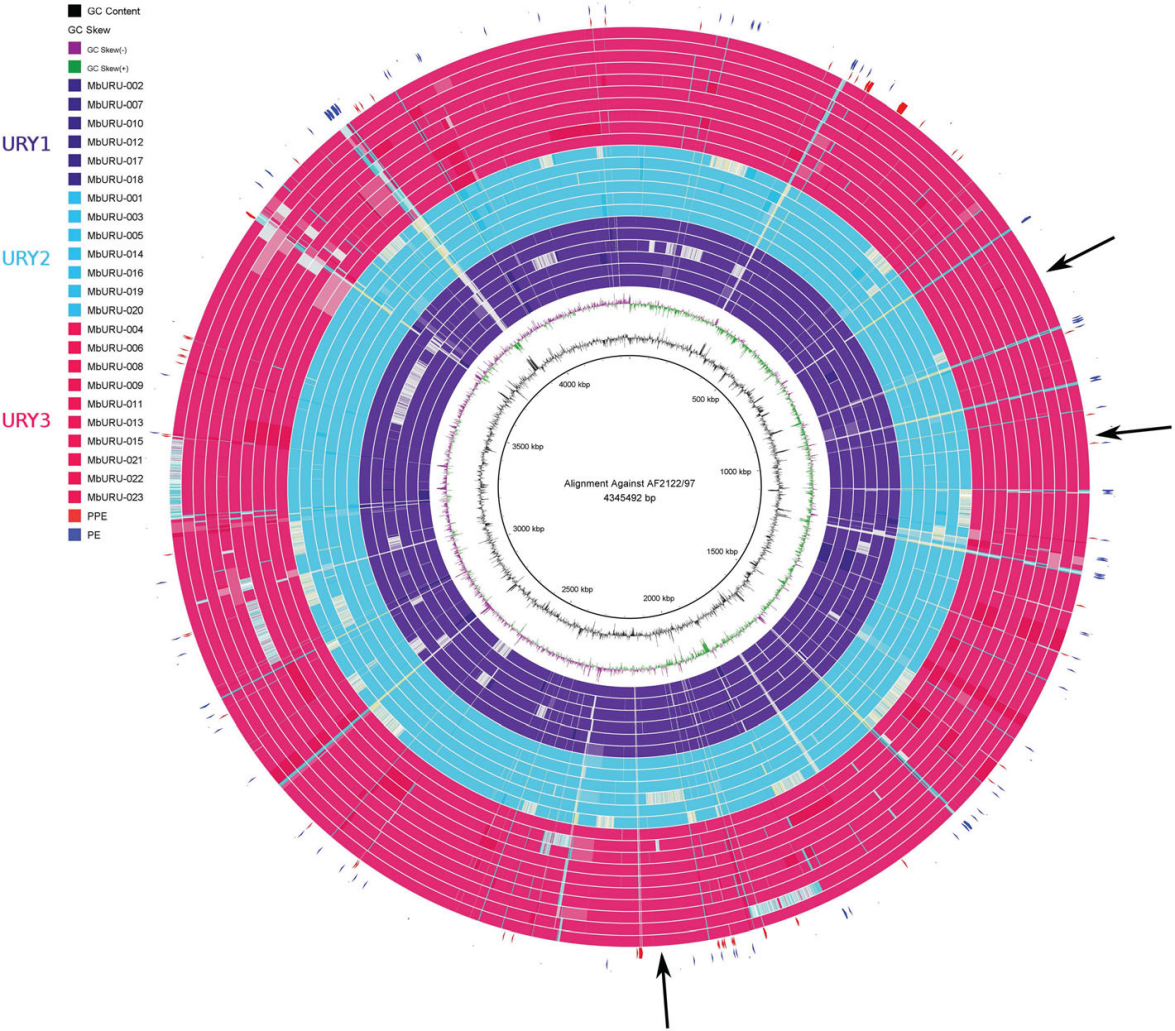
Pairwise alignments of each of the 23 sequenced Uruguayan strains against the reference strain. White regions denote those areas that have identity scores <95% or that are absent in the strain. Blue and red arrowheads indicate the location of gene families PE and PPE, respectively, while black arrows exemplify low identity or absent regions without PE and PPE genes

We delved into the genomic differences among strains which could further explain the observed population structure among the Uruguayan strains. Figure 3 summarizes the genomic variability of the sequenced strains with respect to the reference genome of *M. bovis* [20]. Strains MbURU-012, 014 and 020 exhibit sizable areas of missing information, which can be attributed to the low sequencing depth of their genomes, resulting in smaller genome sizes (3.48 to 4.09 Mbp). To prevent skewing in the data analysis, we defined a minimum genomic coverage of 30X from 100 bp paired end data, for variant analysis, annotation, and other downstream analyses. The scarcity of low identity regions among the local strains is consistent with the proposed 99.9% overall genetic identity among members of the MTBC [20, 21]. Low identity regions identified mainly correspond to genes coding for PE and PPE protein families (blue and red arrowheads in Fig. 3, respectively). Nonetheless, we identified novel areas exhibiting low identity, or even missing, with respect to the reference genome. First, we localized low identity regions, which harbored 25 genes (Additional File 5: Table S3). Among them we found *pks12* and two glycosyltransferases (*Mb1551* and *Mb1553c*) with identity scores between 86% and 93% in nine of the strains. Manual inspection of the alignment shows that several unequivocal mapped reads support these polymorphisms, evidencing that the observed diversity is real. On the contrary, identity scores found in the glycosyltransferases are the result of gene duplications in these strains or the corresponding deletions in the reference strain, such that the observed variability is due to mismapping. (Additional File 6: Figs. S3A-S3B). This is evidenced by the high read coverage of the glycosyltransferases, which reaches two to ten times the genome-wide average in all strains except for MbURU-001, 008 and 021.

Regions of difference (RDs) were identified by inspecting the genome-wide coverage density. We found ten novel RDs are present in the Uruguayan strains when compared against the reference (Table 2). To confirm the validity of these results, we tested the four found to be specific to a given spoligotype (bolded in Table 2). Two of these regions were confirmed by PCR (RDbov130a and RDbov145b, Additional File 7: Figure S4A), and all of them were manually curated by visual inspection of the paired-end read alignments both against the reference genome and against their respective assemblies (Additional File 7: Figure S4B). All strains of spoligotypes SB0130 and SB0145 lack RD3, previously reported as absent in other MTBC strains including *M. bovis*, BCG and *M. tuberculosis* [22]. The remaining nine RDs have not been described previously. Interestingly, many of these regions of difference appear to be spoligotype-specific. For instance, strains with spoligotype SB0130 lack a 994 bp region that comprises the gene *Mb3923c*. Likewise, strains with spoligotype SB0145, share the loss of three different regions, of 604, 546 and 1448 bp, which correspond to the group of genes *Mb0026-Mb0027, xylB* and *Mb0930-Mb0931*, respectively. Other lost regions code for PE and PPE proteins, exhibiting also a spoligotype-specific pattern of loss (Table 2).

**Table 2:** Details of the regions of difference (RD) of lengths greater than 500 bp in the sequenced strains from Uruguay, as well as deletions found in PE/PPE-coding genes. Each RD is defined by an ID, and all deletions show the number of ORFs that cover, starting and ending coordinates, length, strains presenting these deletions and spoligotypes associated with them. Marked in bold are the RDs with more robust association to a given spoligotype

ID		Genes covered	Start	End	Length	Strains (MbURU-)	Spoligotypes associated
Previously described	RD3	14, Mb 1598-Mb1611c	1,764,652	1,773,872	9221	001, 002, 003, 005, 007, 0010, 012, 014, 016	SB0145, SB0130
	RDbov145a	2, Mb0026-Mb0027	29,475	30,078	604	001, 003, 005, 014, 016, 019, 020	SBO145
	RDbovI 31	5, Mb0677c-Mb0681c	756,105	757,654	1550	010	X
	RDbov145b	1, serA2	824,054	824,599	546	001, 003, 005, 014, 016, 019, 020	SB0145
	RDbov145c	2, Mb0930-Mb0931	1,009,480	1,010,927	1448	001, 003, 005, 014, 016, 019, 020	SB0145
	RDbovI 072	7, Mb1908-Mb1914c	2,116,213	2,122,816	6604	006	SB1072
	RDbovI 32	1, rmlB2	3,832,661	3,833,913	1253	007	X
	RDbov133	2, bisC-Mb1478c	1,616,260	1,619,455	3196	012	X
	RDbov134	1, Mb3756	4,115,825	4,116,744	920	012	X
	RDbov130a	1, Mb3923c	4,310,701	4,311,694	994	002, 007, 010, 012, 017, 018	SB0130
PE/PPE		1, PE_PGRS19	1,189,852	1,190,034	183	001, 003, 005, 014, 016, 019, 020	SB0145
		1, PE_PGRS20	1,192,321	1,192,742	422	001, 003, 005, 014, 016, 019, 020	SB0145
		1, PE_PGRS24	1,486,342	1,486,528	187	001, 003, 005, 014, 016, 019, 020	SB0145
		1, PPE30	2,033,505	2,033,624	120	002, 007, 010, 012, 017, 018	SB0130
		1, PE_PGRS42d	2,762,816	2,762,980	165	004, 008, 009, 011, 013, 015, 021, 022, 023	SB0140, SB0274
		1, PE_PGRS50b	3,691,479	3,691,697	219	004, 006, 008, 009, 011, 013, 015, 021, 022, 023	SB0140, SB0274, SB1072
		1, PE_PGRS50b	3,694,231	3,694,311	81	006	SB1072
		1, PE_PGRS51	3,733,072	3,733,247	176	001, 003, 005, 014, 016, 019, 020	SB1045

Duplicated genes in the Uruguayan strains were identified by performing a coverage analysis. A total of 217 duplicated unique genes were identified in all strains (Additional File 8: Table S4). Three of the strains show no duplications (002, 006 and 021), and there are no duplicated genes specifically associated to a given spoligotype. Strain MbURU-018 accounts for 35% (144) of the total set of duplicated genes. In fact, Gene Ontology (GO) analysis of the duplicated genes revealed that only three strains, MbURU-007, 009 and 018, exhibit significant enrichment of duplicated genes. MbURU-007 displays enrichment in the Diterpenoid biosynthesis route. On the other hand, the duplications found in MbURU-009 and 018 enrich for immune system process, defense responses to viruses and defense responses.

We also identified five putative novel genes in the local strains that are not present in the reference genome. One PE_PGRS and one uncharacterized protein are strain specific, present only in MbURU-011 and 015, respectively. The other correspond to novel genes found in more than one strain; an ABC transporter that has been very recently re-cataloged as present in the reference genome after re-sequencing and annotation of AF2122/97 [23], a Ser/Thr protein kinase in 11 of our strains, and a hypothetical protein in strains MbURU-007 and 009 (Additional File 9: Table S5).

To further assess the variability of our strains, we evaluated the presence of polymorphisms. The Uruguayan strains harbor 231 to 551 SNPs, and 18 to 55 insertions/deletions (indels) with respect to the reference genome AF2122/97. Comparative analyses revealed a total of 1366 non-repetitive variants, 499 unique variants and a set of 43 variants common to the 20 strains. As the phylogenetic tree shows in Fig. 2, isolates in group URY3 show less variants than those in groups URY1 and URY2, suggesting these are genetically more akin to the reference strain. URY1, URY2 and URY3 show a set of 161, 296 and 47 unique variants, and 391, 529 and 182 common variants per group, respectively (Table 3).

**Table 3:** Details of the variants found in each of the local strain, separated by spoligotype identity

Group	Spoligotype	Strain (MbURU-)	Total per strain	Unique per strain	Common per group	Unique per group
URY1	SB0130	002	539	8	391	161
		007	456	6		
		010	518	16		
		017	563	50		
		018	511	17		
URY2	SB0145	001	586	10	529	296
		003	592	6		
		005	571	8		
		016	592	16		
		019	578	4		
URY3	SB0274	004	387	9	182	47
		008	316	3		
		009	415	14		
		011	396	11		
		013	249	8		
		015	333	10		
		021	361	5		
		023	329	9		
	SB1072	006	361	85		
	SB0140	022	347	25		

**Fig. 4:**
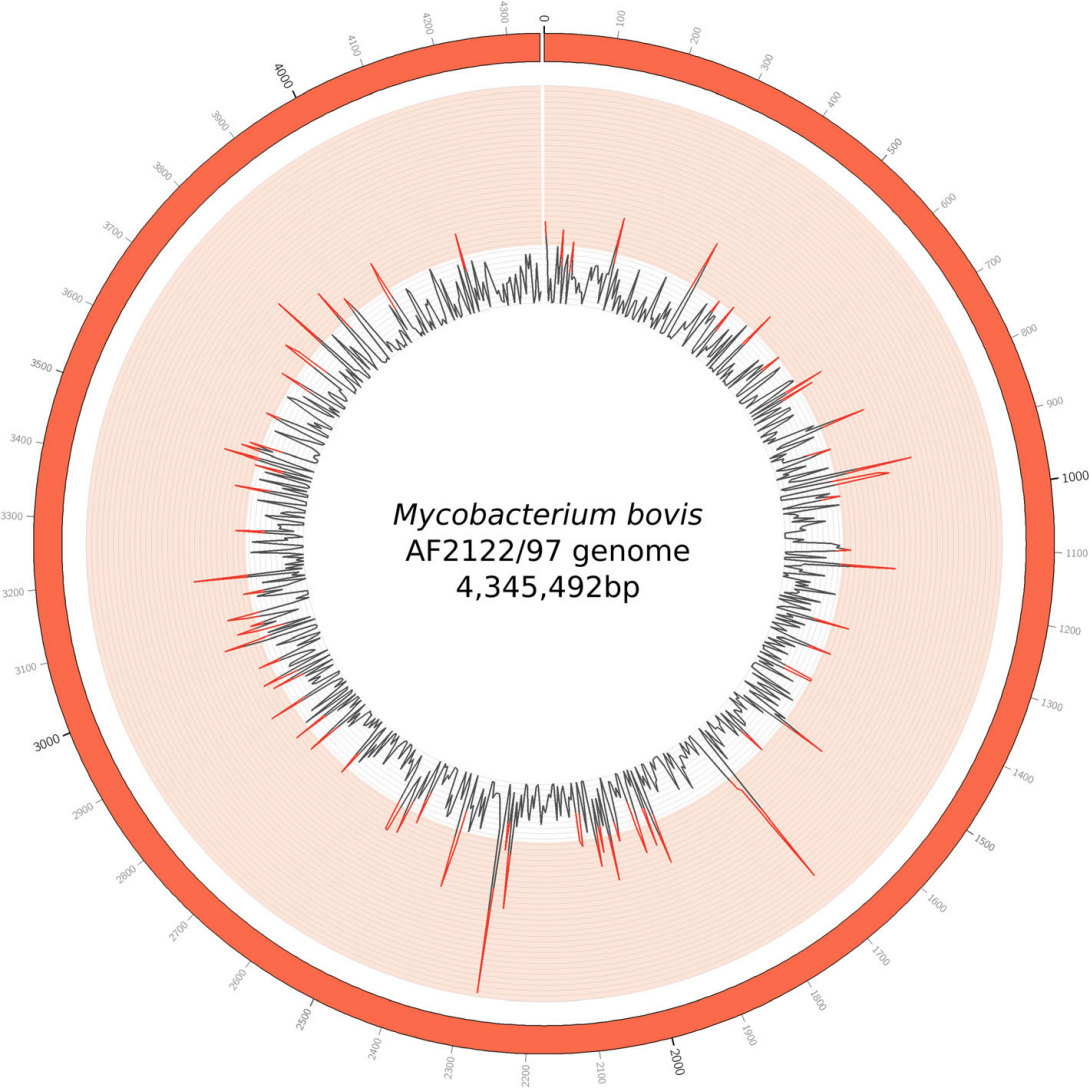
Visualization of the density of variants in the entire length of the reference genome (AF2122/97). Inner rays represent the variant density of every 5 kb region: a region that surpasses a density of 0.006 (innermost circle of the light red band) is significantly high density (binomial test, *p* < 0.001 after Bonferroni correction)

To evaluate the potential existence of SNP clustering in the genome, we calculated the SNP density throughout the genomes in all the Uruguayan strains by establishing a sliding window of 5 kb. The resulting SNP density graph shows a non-random SNP distribution through the genome (binomial test, *p* < 0.001 after Bonferroni correction) with 78 statistically significant regions (Fig. 4, red peaks). Detailed information on these regions (density > 0.006 SNP/kpb) can be found in Additional File 10: Table S6. The genes with low identity values mentioned before, *pks12*, *Mb1551* and *Mb1553c*, had the highest detected SNP density*.*

**Fig. 5:**
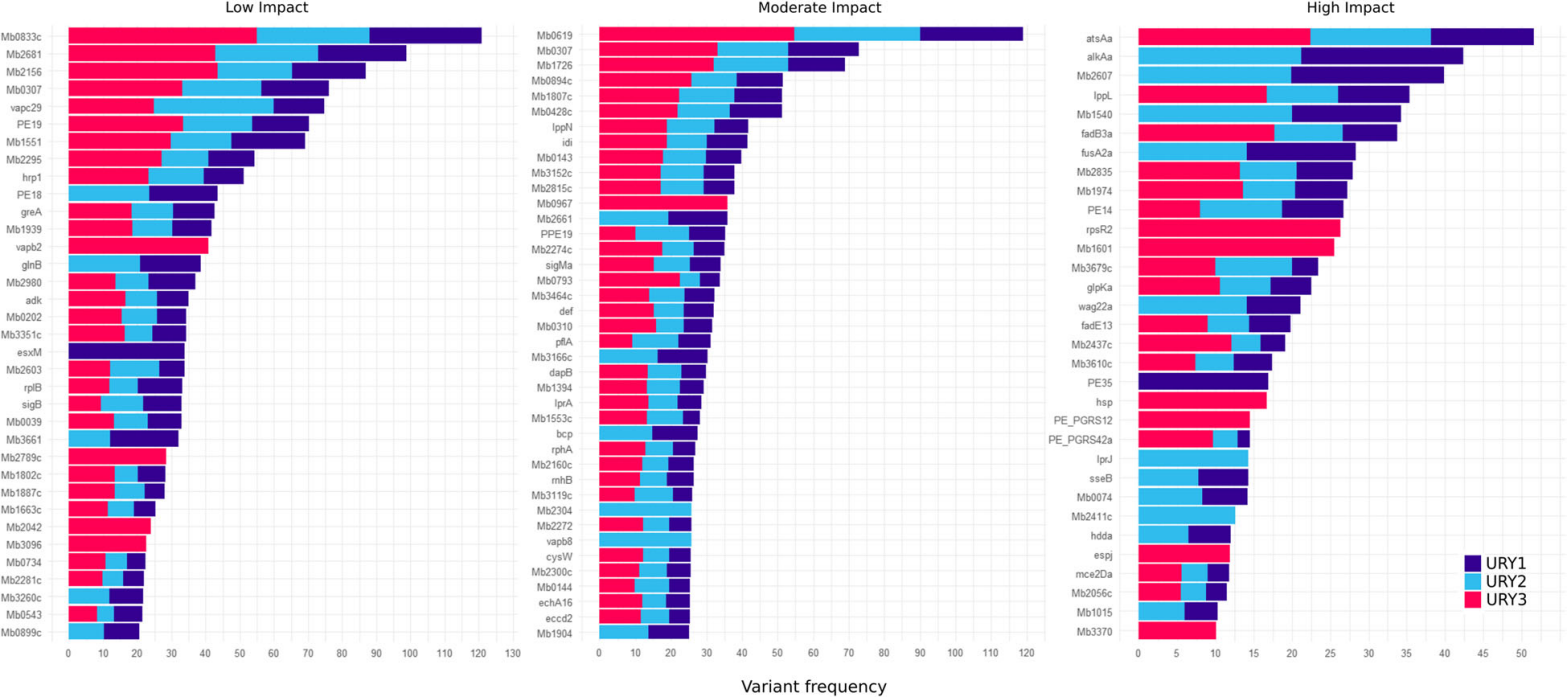
Genes with the highest variant frequency (SNP/kb), categorized according to their impact in the genes as high, moderate and low impact. Each strain is colored according to the group they belong to, as defined by their phylogenetic structure: URY01, URY02 and URY03

We then determined the variant frequency (SNP/kpb) among genes in the 20 high coverage strains, taking into consideration their identities as URY1, URY2 or URY3. Figure 5 shows variants separated in three categories according to their impact on genes (high, moderate and low, as described in [24]). We found 376, 569 and 95 unique genes with low, moderate and high impact variants, respectively. We noted a significant difference in the incidence of variants depending on the group observed.

In order to determine whether genes affected to the same extent by SNPs are involved in common processes we analyzed their GO enrichment for each category (low, moderate and high). While genes with low impact mutations are not enriched in any GO term, we observed some interesting trends for genes moderately and highly impacted.

Genes bearing high impact mutations are not enriched in any particular biological process pathway. While some of these genes are found to have few to no paralogs, others perform functions that are interesting to note. For instance, *Mb0119*, one of the only two annotated sugar kinases in the reference genome of *M. bovis*, exhibits frameshifts in 11 of the studied strains that belong to spoligotypes SB0130 and SB0145. Heat shock protein *hsp* also shows a frameshift in seven of the eight strains of spoligotype SB0274, and the strain belonging to SB0140. Both *iniB* and *iniC*, two of the three genes of the *iniBAC* operon, bear frameshifts in strains. *iniB* appears mutated in strains belonging to spoligotypes SB0274 and SB0130, while frameshifts in *iniC* are only observed for strains of spoligotype SB1072. While we found no frameshifts in the remaining member of the *iniBAC* operon, *iniA*, interestingly, we also found one missense and one synonymous variant for all SB0145 strains. *sppA*, a putative protease IV, possibly involved in the digestion of signal peptides for the secretion of mature proteins across the membrane, was found to have acquired a frameshift in strain 009. Synonymous variants at this gene were also found in strains of the SB0145 and SB1072 spoligotypes. All truncated genes found are shared within a spoligotype, or by population groups. Of particular interest is *plcD*, the only phospholipase C found in *M. bovis*, which was found to be truncated in all strains belonging to spoligotype SB0274. The complete list of truncated genes for these strains can be found in Additional File 11: Table S7.

Genes with moderate impact mutations are enriched in peptidoglycan-based cell wall biogenesis and carbohydrate metabolic processes. All studied strains contribute equally to the enrichment of these terms, whereby strains from URY2 bear the highest number of genes associated to these terms (Additional File 12: Figure S5).

PE and PPE families of proteins represent a major source of antigenic variability in MTBC members [25–27], Notably, even though PE and PPE families are known for their high variability among strains due to their strong association to pathogenicity [28], their coding genes do not display a high frequency of variants relative to the total set of affected genes. Of the 136 known PE/PPE genes, 58 were found to show at least one variant with respect to the reference, and only 22 show a mutation rate higher than the mutation average for all affected genes (9.43 variant/kbp).

## Discussion

Assessing the existing diversity of *M. bovis* strains and its correlation to pathogenicity and severity of disease is of paramount importance to the economic growth and public health in Uruguay, where cattle outnumber people by almost four to one, and the cattle industry is a major contributor to the country’s GDP. In this study, we report the genome structure of 23 novel Uruguayan *M. bovis* isolates, and compare them to the reference strains.

Currently, four main clonal complexes of *M. bovis* have been described, European 1 (Eu1) [13], European 2 (Eu2) [14], African 1 (Af1) [12], and African 2 (Af2) [11]. The 23 Uruguayan strains sequenced here show spoligotype patterns lacking spacer 11, concordant with strains from Eu1, which were originally identified throughout Britain and Ireland as well as former British colonies (South Africa, Australia, New Zealand, Canada and the United States), and Latin America with the exception of Brazil [10]. This clonal complex is rare in mainland Europe [13], a region dominated by Eu2 which is also the most frequent in Brazil [14]. Af1 and Af2 clonal complexes are restricted to countries from West-Central and East Africa, respectively [10, 29].

Although all Uruguayan strains belong to Eu1, we identified three highly structured groups (URY1, URY2, and URY3), showing high genetic diversity among them, consistent with the genomic variability reported in this work. The estimated diversity statistics (i.e. π and θW) for synonymous sites revealed an excess of rare alleles (θW > π) in URY1 and URY2, but not in URY3, indicating a deviation from neutrality due to demographic processes and/or population structure. For non-synonymous sites, π and θW values are not significantly different.

Eu1 likely reached its current global distribution with the trade of modern British cattle breeds, perhaps within the last 200 years [10]. The high genetic diversity found among Uruguayan *M. bovis* can probably be attributed to the extensive history of British cattle breeding in the country, and the lack of clear geographic barriers separating Uruguay from Brazil.

These three highly structured groups were congruently devised both by core genome and variant calling analyses. URY1 represents a genetically more heterogeneous group than URY2 and URY3. At the same time, these last two show closer genetic similarity between them. When compared to the reference, groups URY1 and URY2 present a higher number of variants than URY3, and a differential pattern of variant incidence between these two sets is evidenced. This clustering was also evidenced by the absence of region of difference RD3 in URY1 and URY2, which contains ORFs from phiRv1 prophage, present in URY3. Strains from URY3 are more closely related to the reference strain of *M. bovis,* AF2122/97 (SB0140) [20], than to the remaining strains. Spoligotype SB0140 is known to be a predecessor of spoligotypes SB0274 and SB1072. This is in accordance with our phylogeny of SNPs and indels, where the reference shares more variants with strains from group URY3 than with the other two. The complete set of distinctive polymorphisms for the three groups can be found in Additional File 13: Table S8.

Our comparative analysis revealed a set of genomic traits that begin to explain the observed strain clustering and/or are informative as sources of variability previously unknown. PE and PPE families of proteins represent a major source of antigenic variability in MTBC members [25–27], but a small amount of variable areas that do not belong to these families represent useful sources of variability for the characterization of these strains. Among these are RDs which cover up to seven ORFs, most of which are annotated as conserved hypothetical proteins. The fact that some RDs are found to be absent in all strains of a given spoligotype leads us to propose that spoligotypes, which focus on a very small region of the genome of MTBC [30], can be complemented by analyzing the absence of specific RDs to localize lineages and sub lineages. While spoligotyping by itself is ineffective for phylogenetic applications given its high homoplasy rate [16], it is a good epidemiological method when used along with other techniques such as MIRU-VNTR [31]. The idea of complementing these markers with large sequence polymorphisms adjusts itself harmoniously with the suggestion that particular “signature” spoligotyping patterns can be indeed informative for population genetic analyses, where many strains can be grouped using such “signature” patterns [16]. RD typing has had a widespread use in resolving phylogenetic relationships, and it was later succeeded by SNP typing. It has also been used as a companion approach to SNP calling for predictive purposes, in search for the development of new SNP-typing barcodes [32]. Identifying genomic features like RDs that are specific to sub lineages represents a useful source of information to delineate the fine differences among strains of identical spoligotype patterns, which our data suggests do not share a common ancestor. This, taken together with our results, supports that RDs are good markers for sub lineages and should therefore not be left aside. IDs shown in bold in Table 2 indicate RDs suggested for use as markers to characterize the spoligotypes found in our samples: RD145a, RD145b and RD145c and RD130a.

SNPs are also a contributing factor to the variability of some regions, including several of the low identity genes found in these strains: the glycosyltransferases *Mb1551* and *Mb1553c,* and pks12, the longest gene in bTB and tuberculosis genomes coding for a polyketide synthase implicated in antigenic variation [33]. Of these three, lower identities in the glycosyltransferases can be explained by their exceptionally high read coverage in all but three of our strains. This hints at duplicated genes, that the assembler collapsed, which were consequently perceived as bearing low identity to the reference in strains MbURU-002, 003, 007, 009, 011, 016, 022 and 023. A high degree of diversity was found among the identified duplicated genes. Particularly, strains 009 and 018 have an exceptionally high number of duplications relative to the other strains. Large numbers of duplicated genes could be associated with a genomic adaptation to a changing environment by means of a gene dosage effect. It has been proposed before that while gene duplication might not necessarily double gene dosage, due to potential negative feedback loops, it generally leads to its increase [34–36]. Biological assays will be required to determine whether a functional relationship exists between this genomic feature and the enriched biological processes of defense response to virus and immune system.

An average of 131 (36%) synonymous and 229 (64%) non-synonymous SNPs were found among our strains. The genomes present a high dN/dS ratio, which is consistent with previous assertions of low levels of purifying selection in MTBC members [37]. We found a bias in moderate impact variants towards genes involved in cell wall organization pathways, as it is the case for *iniBAC* promoter genes, which are induced by inhibitors of cell wall biosynthesis. At the same time, this enrichment is not apparent when the strains are studied individually. There seems to be a lack of a consensus strategy for cell wall organization, which implies different means to the same end. Heterogeneity of cell surfaces is an adaptive trait among populations of pathogenic bacteria, which allows different sub populations to thrive in a variety of environments within the host. This would allow the organisms to adhere to a greater variety of surfaces, putting cell wall component genes under diversifying selection [38, 39]. On the other hand, high impact variants in our strains affected many genes with unique or noteworthy functions, however, no bias in biological pathways were identified. Further analysis of the functional consequences of these mutations and examination of these in larger sample numbers is required. The number of variants in PE and PPE genes drew a different picture for this family. Unexpectedly, most members of this family do not present a higher frequency of variants than the average. On one hand, many of these genes are known to exhibit extensive genetic variation, with many mechanisms having been reported that contribute to this [28]. On the other hand, the main mechanism of variation within these families could be events of homologous recombination that result in deletions or duplications of whole regions in the genes, instead of a high frequency of variants. This is highly likely, given that most members in these families contain domains comprised of series of tandem repeats, making them more prone to undergo recombination [40].

The analysis presented here sheds light onto *M. bovis* genomic variability worldwide, as well as**,** the nature of Uruguayan strains. Moreover, it supports the existence of a larger pattern of genomic variability than that provided by a spoligotype classification. In small populations, spoligotypes are good indicators of the genomic similarity among strains, as it is evidenced by the spoligotype/SNP correlation observed in the Uruguayan strains. However, for the purposes of comparison among multiple populations, in a worldwide context, spoligotypes fall short. High homoplasy rates blur the initial correlation between these two markers evidencing the clear need to resort to additional typing means. RDs have shown to be genomic markers with good resolution in defining sub-lineages and we therefore proposed them as appropriate markers. The incorporation of meta data will also be crucial for a more thorough characterization of the Uruguayan population of *M. bovis,* allowing a definitive association between all genomic traits described here and their potential phenotypic effects.

## Conclusions

We have sequenced and analyzed 23 genomes of *M. bovis* in Uruguay. Comparative studies of these strains showed that, while they all belong to the clonal complex European 1, they exhibit a surprisingly structured phylogenetic tree, and a high level of genomic variability. Localization of the genomic sources of variability for Uruguayan strains was attained. We observed duplicated genes with high diversity among strains, differential distribution of regions of difference, distinct SNP patterns of incidence which specifically grouped with spoligotype patterns, novel genes absent in the reference, and variable genes. In future studies, it will be interesting to assess the association of this high variability of such genomic features with phenotypical traits in order to gain insights into the functional consequences of this diversification.

## Methods

### *M. bovis* Strains

All samples were granted by Dirección General de Servicios Ganaderos (DGSG), obtained during routine surveillance and eradication campaigns conducted in slaughter plants.

#### Phylogenetic reconstruction of 186 MTBC strains

The 164 MTBC strains used to compare the *M. bovis* strains from Uruguay were downloaded from public databases, assembled with SPAdes [41], and annotated with Prokka [42]. The pan genome of all the 186 strains was estimated with Roary (v3.6.8) [43]; core genes were defined as those present in all 186 isolates with a 90% ID cut-off. Recombination was inspected using fastGEAR [44]. Finally, core genes aligned with Mafft [45] were used to reconstruct the phylogenetic relationships by maximum likelihood using RAxML [46], with GTRCAT model and autoMRE for bootstraping. For each Uruguayan group (i.e. URY1, URY2, URY3), two measures of genetic diversity were estimated, the average pairwise nucleotide diversity per site (π, [47]) and Watterson’s θ (θW), which is based on the number of segregating sites [48]. For both measurements, synonymous and non-synonymous diversity was calculated separately using the R package PopGenome [49].

#### Strain selection, culture and DNA extraction

23 *M. bovis* strains were used for whole genome sequencing, obtained from ten departments of Uruguay in the years 1982, 1998, 2005, 2008, 2010, 2014 and 2015. Samples were decontaminated using the 5% oxalic acid decontamination method [50], using equal parts of both decontaminant and sample for 15 min at 37 °C. Tissues were homogenized (Stomacher 400) and later centrifuged at 2800 rpm for 30 min. The resulting sediments were inoculated on Löwenstein-Jensen and Stonebrink media, incubated at 37 °C and screened weekly for macroscopic growth until eight weeks. Identification of mycobacteria was based on observation of smears submitted to the Ziehl-Neelsen method, growth characteristics such as time, temperature and colony morphology. Suspected colonies were then evaluated with biochemical tests [51]. Genomic DNA was purified from supernatants of the strains diluted in TE (Tris-EDTA) and warmed at 100 °C for 10 min. Experimental corroboration of the extracted DNA as belonging to *Mycobacterium tuberculosis* complex (MTBC) was performed by PCR of the ETR-D fragment as previously described [52].

#### Whole genome sequencing and typification

Sequencing of the 23 strains was performed at the Institut Pasteur de Montevideo on an Illumina MiSeq platform from a paired-end library (2 × 75 cycles). Briefly, Nextera XT (Illumina, USA) library preparation kit was used from 1 ng of total DNA according to manufacturer instructions. Index primers were added to each library to allow sequence multiplexing. After 12 PCR cycles, the final library was purified with AMPure XP (Benchman, USA) and quantified with the Qubit dsDNA HS assay kit (Invitrogen, USA). Quality and length of the librarywere assessed with the Agilent high-sensitivity DNA kit (Agilent, USA) using the 2100 Bioanalyzer (Agilent, USA). Quality assessment of the resulting reads was performed using NGSQCToolkit (v2.3.3) [53]. Those reads with overall quality score below 20 were filtered out. From the remaining reads, we calculated the resulting coverage of each genome. If the coverage was lower than 30X, we rejected the whole sequencing project. This value was chosen to ensure a good quality of the variants called in downstream analysis. Typification of all strains was performed in silico with the tool SpolPred [54]. SpolPred predicts the spoligotype pattern of a strain based on the reads of a whole genome sequencing project.

#### Genome assembly

Velvet (v1.2.10) [55] was used to perform a de novo assembly of the local strains. Multiple independent assemblies were performed for each strain, differing in the chosen k-mer value, which ranged from 17 to 61. We chose the best k-mer and respective assembly based on the amount of resulting contigs, the N50 and the relation between the overall length of the assembly and the sum of both the lengths of the contigs under and over than 1000 bp. We expect a lower amount of contigs with a high N50 and an overall assembly length close to the known length of the reference strain AF2122/97. We also performed two iterations of confirmatory assembly using SPAdes (v3.6.1) [41], using the previous Velvet assembly as known data and changing the set of trial k-mers in each. All three resulting assemblies were integrated with the software CISA (v1.3) [56].

#### Genome improvement

PAGIT toolkit [57] was used to further improve the quality of the assembly by closing gaps on scaffolds, correcting base errors and generate an annotation of CDS based on the reference genome AF2122/97. The final assembly was also automatically annotated with RAST server [58] and Prokka [42]. tRNA and rRNA genes were identified with ARAGORN [59] and barnap (v0.6), respectively.

#### Genome alignment against the reference

Genome comparison of the Uruguayan strains relative to the reference was done using BLAST Ring Image Generator (BRIG) software [60], which performed pairwise alignments for the 23 genomes. Chosen identity values to be displayed were 98% and 95% as upper and lower identity threshold, respectively.

#### Mapping to the reference and coverage analyses

Paired-end reads were first aligned to the GRCh38 human genome assembly (GenBank accession GCF_000001405.28) with BWA (v0.7.12-r1039) [61], in order to filter out possible human sequenced reads resulting from manipulation errors in the sequencing stage. From the remaining reads, we then performed a second mapping to the reference strain *M. bovis* AF2122/97 (GenBank accession NC_002945), allowing up to 3 mismatches in each seed of length 15 bp. Samtools mpileup [62] was ran on the mapped reads to get the coverage at each base. This was used to identify RDs and duplicated genes. For the former we extracted those regions with coverage 0 and a length higher than 500 bp. For the latter, we qualified as duplicated genes those whose median coverage was higher than 2 in more than 70% of the length of each gene.

#### Validation of regions of difference

To verify the predicted RDs we used two strategies. Firstly, by PCR. The PCR reactions targeted at a region inside each RD consisted of Mango-Taq (Bioline, London, UK), primers listed in Additional File 7: Figure S4A (Integrated DNA Technologies), and the following cycling parameters: 4 min at 95 °C, 37 cycles at 94 °C for 30 s, at 55 °C for 20 s and 72 °C for 30 s, with 4 min at 72 °C for the final extension. We analyzed the reaction results on a 1% agarose gel and determined whether the primers amplified or not (showing absence or presence of the RD, respectively), or if the results were not conclusive (True, False and Inconclusive in Additional File 7: Figure S4A). Secondly, we further investigated the regions by mapping and performing manual inspection of the sequenced reads mapped to the reference and the reads mapped to the assembled genome though the Integrative Genomics Viewer (IGV, [63]). If the RD was an artifact, the number of mapped reads would slowly decrease before reaching zero. Furthermore, we analyzed the insert sizes of those reads flanking the absent regions, where we expected red-colored reads (with insert sizes larger than expected) flanking the RDs in an alignment against the reference genome. Conversely, mapping against the assembled genomes should not is not expected to show blue-colored reads (insert sized smaller than expected). Finally, we assessed the support of reads in the position where the deletion would occur.

#### Novel gene prediction

From the mapping to the reference performed with BWA, we kept the unaligned reads of the 23 Uruguayan strains and assembled each subset de novo with Velvet. RAST was used to find ORFs from the resulting contigs and annotate them. We filtered out those sequences that were smaller than 225 nucleotides and performed a blastp of all remaining sequences against each other to find common novel genes between strains and against the NCBI database to locate the highest identity matches against them.

#### GO annotation

The Gene Ontology (GO) terms for any set of genes were analyzed as follows. The orthologous genes for each gene in *M. tuberculosis* H37Rv were obtained by reciprocal blastp. Given the high identity between these two members of MTBC, there was no need for obtaining orthologous genes by alternative methods. For this set of genes, we acquired the prioritized biological process GO terms (*p* < 0.05) and their fold enrichment from the Gene Ontology project [64].

#### Variant calling and clustering

The reads and pairs that mapped to the reference were filtered in with Samtools to later perform variant calling (v0.1.18) [62], not including the three low coverage strains (< 30X). Samtools mpileup and VarScan (v2.3.7) [65] were used for variant calling, filtering both indels and SNPs with a minimum of 20 supporting reads at a position to call variants (−-min-reads2) and a minimum variant allele frequency threshold of 0.2 (−-min-var.-freq). In order to cluster the local strains and visualize their relatedness we performed a principal component analysis (PCA) from the variants of the 23 Uruguayan strains using the package *adegenet* [66]. A maximum likelihood phylogeny was estimated from these variants using RAxML (v8.2.7) with GTRGAMMA model and 100 bootstrapping iterations [46], choosing *M. caprae* as outgroup (SRA Accession: SRR1792164). From the SNPs identified, we also calculated the SNP density along the length of the reference strain AF2122/97 using a sliding window of 5 kb (SNP absolute frequency divided by the length of the window). To visualize if there were heterogeneous SNP-dense regions in the genome, we visualized these densities with Circos (v0.69) [67]. We used SnpSift (v4.2) [68], a vcf-manipulation tool, to extract all the genes contained between the SNP-densest regions.

#### SNP annotation

SnpEff was used to annotate variants of the local strains (v4.2, build 2015–12-05) [24], classifying them as synonymous or non-synonymous and obtaining their respective impact according to their incidence in the resulting gene (high, medium, low. See SnpEff documentation). Based on this information, we also calculated the number of variants each gene showed for each strains divided by the gene length in kilobases as a relative measure of SNPs (SNP/kb). Finally, we performed a GO search to obtain the most affected terms.

## Additional files

Additional file 1: Figure S1. Sequencing and genome annotation statistics for the 23 Uruguayan strains of *M. bovis*. (PDF 115 kb)

Additional file 2: Table S1. *M. bovis* strains isolated from a bovine host in Uruguay. (ODS 26 kb)

Additional file 3: Figure S2. List of tRNAs and corresponding codons in the Uruguayan genomes under study. (PDF 66 kb)

Additional file 4: Table S2. Details of the 163 MTBC strains downloaded from NCBI and the 23 Uruguayan strains from this study, used to resolve a phylogenetic tree out of their core genomes. (ODS 34 kb)

Additional file 5: Table S3. Resulting blastn of all non-pe/ppe coding sequences against *M. bovis* AF2122/97. (ODS 24 kb)

Additional file 6: Figure S3a. Interactive Genome Visualizer (IGV) visualization of reads aligned to reference genome AF2122/97 (exemplified by strains MbURU-002, MbURU-003 and MbURU-010), showing a region containing glycosyltransferase-coding genes Mb1551 and Mb1553c (wbbL2). Coverage is represented in the upper track of each strain as a gray bar chart, and SNPs are showed as colored bars. The average coverage for these genes can reach up to 10 times the average genome coverage. **Figure S3b.** IGV visualization of reads from strain MbURU-002 aligned to reference genome AF2122/97, showing a region containing polyketide synthase *pks12* gene. The lower alignment represents only reads with mapping qualities higher than 0 and show therefore no multimapping reads, while the upper alignment represents the original data from MbURU-002. Coverage is represented in the upper track of each strain as a gray bar chart, and SNPs are showed as colored bars. Note that there are very few called SNPs in this region and some are still present once multimapping reads have been removed. (PDF 2630 kb)

Additional file 7: Figure S4a. Validation of in silico RD typing with PCR on 21 of the Uruguayan *M. bovis* strains. **Figure S4b.** Alignment of the sequenced reads of strain MbURU-003 against the assembled genome of the same strain. Selected pair of reads in red exemplify one of the reads that flanks both sides of a region of difference (RDbov145a) that is absent in this strain. (PDF 1643 kb)

Additional file 8: Table S4. List of the over represented genes found in the 23 Uruguayan *M. bovis* strains. (ODS 18 kb)

Additional file 9: Table S5. Putative novel genes identified in the Uruguayan strains by assembly of the unmapped reads. (ODS 14 kb)

Additional file 10: Table S6. Regions with high SNP density. (ODS 24 kb)

Additional file 11: Table S7. List of truncated genes for the Uruguayan strains studied. (ODS 20 kb)

Additional file 12: Figure S5. Bar plots displaying the number of genes affected per strain that are associated to the GO terms carbohydrate metabolic process and peptidoglycan-based cell wall biogenesis. (PDF 1342 kb)

Additional file 13: Table S8. Details on the distinctive characteristics defining each of the three groups described in this study: URY1, URY2 and URY3. (ODS 250 kb)

## Abbreviations

Af1: African 1 clonal complex
Af2: African 2 clonal complex
bTB: Bovine tuberculosis
Eu1: European 1 clonal complex
Eu2: European 2 clonal complex
GDP: Gross domestic product
GO: Gene Ontology
MIRU-VNTR: Mycobacterial interspersed repetitive unit-variable number of tandem repeat
MTBC: *Mycobacterium tuberculosis* complex
ORF: Open Reading Frame
PCA: Principal Component Analysis
RD: Region of difference
SNP: Single nucleotide polymorphism
