# Supplementary figures and images for: Whole genome sequencing of the monomorphic pathogen Mycobacterium bovis reveals local differentiation of cattle clinical isolates
Source: BMC Genomics. 2018 Jan 2;19:2. doi: 10.1186/s12864-017-4249-6 (PMC5748942; doi:10.1186/s12864-017-4249-6)

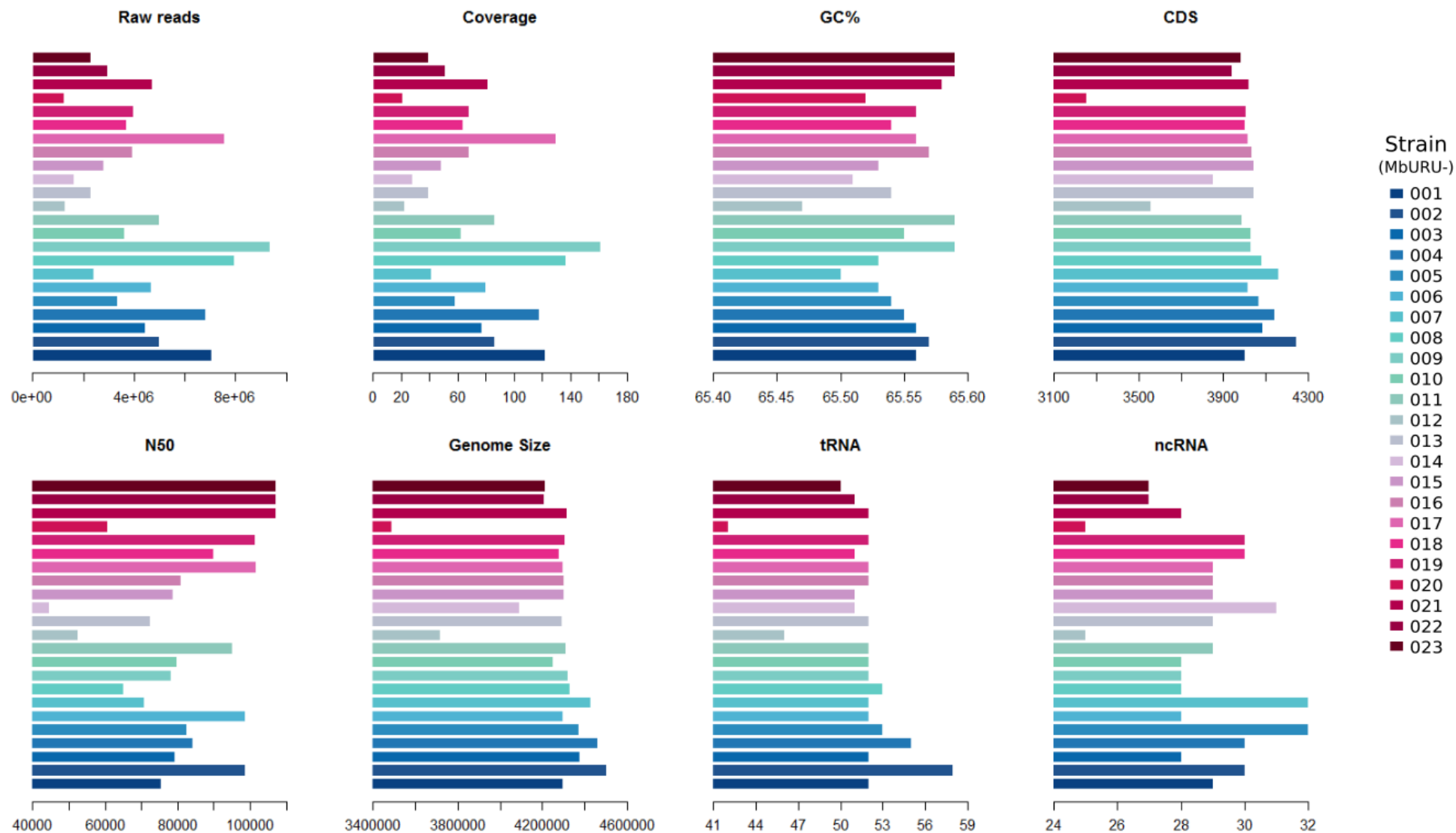

**Figure S1.-** Sequencing and genome annotation statistics for the 23 Uruguayan strains of *M. bovis*.

Supplement: Supplementary file 1 — Sequencing and genome annotation statistics for the 23 Uruguayan strains of M. bovis. (PDF 115 kb) [file 12864_2017_4249_MOESM1_ESM.pdf]
