# Supplementary material for: Whole genome sequencing of the monomorphic pathogen Mycobacterium bovis reveals local differentiation of cattle clinical isolates
Source: BMC Genomics. 2018 Jan 2;19:2. doi: 10.1186/s12864-017-4249-6 (PMC5748942; doi:10.1186/s12864-017-4249-6)
Supplement: Supplementary file 3 — List of tRNAs and corresponding codons in the Uruguayan genomes under study. (PDF 66 kb) [file 12864_2017_4249_MOESM3_ESM.pdf]

■ - Absence
X - Presence

		Strain (MbURU-)																						
		001	002	003	004	005	006	007	008	009	010	011	**012**	013	**014**	015	016	017	018	019	**020**	021	022	023
Ala	tgc	X	X	X	X	X	X	X	X	X	X	X	X	X	X	X	X	X	X	X	X	X	X	X
	ggc	X	X	X	X	X	X	X	X	X	X	X	X	X	X	X	X	X	X	X	X	X	X	X
		X	X	X	X	X	X	X	X	X	X	X	X	X	X	X	X	X	X	X	X	X	X	X
	cgc	X	X	X	X	X	X	X	X	X	X	X	X	X	X	X	X	X	X	X		X	X	X
Arg	acg	X	X	X	X	X	X	X	X	X	X	X	X	X	X	X	X	X	X	X	X	X	X	X
	cct	X	X	X	X	X	X	X	X	X	X	X	X	X	X	X	X	X	X	X	X	X	X	X
	ccg	X	X	X	X	X	X	X	X	X	X	X	X	X	X	X	X	X	X	X	X	X	X	X
		X	X	X	X	X	X	X	X	X	X	X	X	X	X	X	X	X	X	X	X	X	X	X
																					X			
	tct	X	X	X	X	X	X	X	X	X	X	X	X	X	X	X	X	X	X	X		X	X	X
Asn	gtt	X	X	X	X	X	X	X	X	X	X	X	X	X	X	X	X	X	X	X	X	X	X	X
Asp	gtc	X	X	X	X	X	X	X	X	X	X	X	X	X	X	X	X	X	X	X	X	X	X	X
Cys	gca	X	X	X	X	X	X	X	X	X	X	X	X	X	X	X	X	X	X	X	X	X	X	X
Gln	ctg	X	X	X	X	X	X	X	X	X	X	X	X	X	X	X	X	X	X	X	X	X	X	
					X																			
	ttg	X	X	X	X	X	X	X	X	X	X	X	X	X	X	X	X	X	X	X		X	X	X
Glu	ctc	X	X	X	X	X	X	X	X	X	X	X	X	X	X	X	X	X	X	X	X	X	X	
					X																			
	ttc	X	X	X	X	X	X	X	X	X	X	X	X	X	X	X	X	X	X	X	X	X	X	X
Gly	ccc	X	X	X	X	X	X	X	X	X	X	X	X	X	X	X	X	X	X	X	X	X	X	X
	tcc	X	X	X	X	X	X	X	X	X	X	X	X	X	X	X	X	X	X	X		X	X	X
	gcc	X	X	X	X	X	X	X	X	X	X	X	X	X	X	X	X	X	X	X	X	X	X	X
		X	X	X	X	X	X	X	X	X	X	X	X	X	X	X	X	X	X	X	X	X	X	X
			X																					
His	gtg	X	X	X	X	X	X	X	X	X	X	X	X	X	X	X	X	X	X	X	X	X	X	X
		X	X	X	X	X	X	X	X	X	X	X		X	X	X	X	X	X	X	X	X	X	X
									X															
Ile	gat	X	X	X	X	X	X	X	X	X	X	X	X	X	X	X	X	X	X	X	X	X	X	X
Leu	caa	X	X	X	X	X	X	X	X	X	X	X	X	X	X	X	X	X	X	X		X	X	X
	taa	X	X	X	X	X	X	X	X	X	X	X	X	X		X	X	X	X	X	X	X	X	X
			X		X																			
	tag	X	X	X	X	X	X	X	X	X	X	X	X	X	X	X	X	X	X	X		X	X	X
	aag	X	X	X	X	X	X	X	X	X	X	X	X	X	X	X	X	X	X	X		X	X	X
	gag	X	X	X	X	X	X	X	X	X	X	X	X	X	X	X	X	X	X	X		X	X	X
			X																					
Lys	ttt	X	X	X	X	X	X	X	X	X	X	X	X	X	X	X	X	X	X	X	X	X	X	X
	ctt	X	X	X	X	X	X	X	X	X	X	X	X	X	X	X	X	X		X	X	X	X	X
Met	cat	X	X	X	X	X	X	X	X	X	X	X	X	X	X	X	X	X	X	X	X	X	X	X
		X	X	X	X	X	X	X	X	X	X	X	X	X	X	X	X	X	X	X	X	X	X	X
		X	X	X	X	X	X	X	X	X	X	X		X	X	X	X	X	X	X	X	X	X	X
Phe	gaa	X	X	X	X	X	X	X	X	X	X	X	X	X	X	X	X	X	X	X	X	X	X	X
Pro	cgg	X	X	X	X	X	X	X	X	X	X	X		X	X	X	X	X	X	X	X	X	X	X
	tgg	X	X	X	X	X	X	X	X	X	X	X	X	X	X	X	X	X	X	X		X	X	X
															X									
	ggg	X	X	X	X	X	X	X	X	X	X	X	X	X	X	X	X	X	X	X	X	X	X	X
		X	X	X	X	X	X	X	X	X	X	X		X			X	X	X	X		X		X
Ser	cag	X	X	X	X	X	X	X	X	X	X	X	X	X	X	X	X	X	X	X	X	X	X	X
	gga	X	X	X	X	X	X	X	X	X	X	X		X	X	X	X	X	X	X	X	X	X	X
			X																					
	cga	X	X	X	X	X	X	X	X	X	X	X	X	X	X	X	X	X	X	X	X	X	X	X
			X																					
	gct	X	X	X	X	X	X	X	X	X	X	X	X	X	X	X	X	X	X	X	X	X	X	X
	tga	X	X	X	X	X	X	X	X	X	X	X	X	X	X	X	X	X	X	X		X	X	X
Thr	tgt	X	X	X	X	X	X	X	X	X	X	X	X	X	X	X	X	X	X	X	X	X	X	X
	ggt	X	X	X	X	X	X	X	X	X	X	X	X	X	X	X	X	X	X	X	X	X	X	X
		X	X	X	X	X	X	X	X	X	X	X		X	X	X	X	X	X	X	X	X	X	X
	cgt	X	X	X	X	X	X	X	X	X	X	X	X	X	X	X	X	X	X	X	X	X	X	X
						X																		
Trp	cca	X	X	X	X	X	X	X	X	X	X	X	X	X	X	X	X	X	X	X	X	X	X	X
Tyr	gta	X	X	X	X	X	X	X	X	X	X	X	X	X	X	X	X	X	X	X	X	X	X	X
			X																					
Val	tac	X	X	X	X	X	X	X	X	X	X	X	X	X	X	X	X	X	X	X	X	X	X	X
	cac	X	X	X	X	X	X	X	X	X	X	X	X	X	X	X	X	X	X	X	X	X	X	X
	gac	X	X	X	X	X	X	X	X	X	X	X	X	X	X	X	X	X	X	X	X	X	X	X
tmRNA	ADSHQRDYALAA*	X	X	X	X	X	X	X	X	X	X	X	X	X	X	X	X	X	X	X	X	X	X	X
	**Total tRNA:**	**52**	**58**	**52**	**55**	**53**	**52**	**52**	**53**	**52**	**52**	**52**	**46**	**52**	**51**	**51**	**52**	**52**	**51**	**52**	**42**	**52**	**51**	**50**

Figure S2.- List of tRNAs and corresponding codons in the Uruguayan genomes under study. Strains MbURU-012, 014 and 020 (in bold) showed lower genome sequencing coverage and therefore fewer tRNAs were annotated (see text).
