# Supplementary material for: Whole genome sequencing of the monomorphic pathogen Mycobacterium bovis reveals local differentiation of cattle clinical isolates
Source: BMC Genomics. 2018 Jan 2;19:2. doi: 10.1186/s12864-017-4249-6 (PMC5748942; doi:10.1186/s12864-017-4249-6)
Supplement: Supplementary file 6 — Interactive Genome Visualizer (IGV) visualization of reads aligned to reference genome AF2122/97 (exemplified by strains MbURU-002, MbURU-003 and MbURU-010), showing a region containing glycosyltransferase-coding genes Mb1551 and Mb1553c (wbbL2). Coverage is represented in the upper track of each strain as a gray bar chart, and SNPs are showed as colored bars. The average coverage for these genes can reach up to 10 times the average genome coverage. Figure S3b. IGV visualization of reads from strain MbURU-002 aligned to reference genome AF2122/97, showing a region containing polyketide synthase pks12 gene. The lower alignment represents only reads with mapping qualities higher than 0 and show therefore no multimapping reads, while the upper alignment represents the original data from MbURU-002. Coverage is represented in the upper track of each strain as a gray bar chart, and SNPs are showed as colored bars. Note that there are very few called SNPs in this region and some are still present once multimapping reads have been removed. (PDF 2630 kb) [file 12864_2017_4249_MOESM6_ESM.pdf]

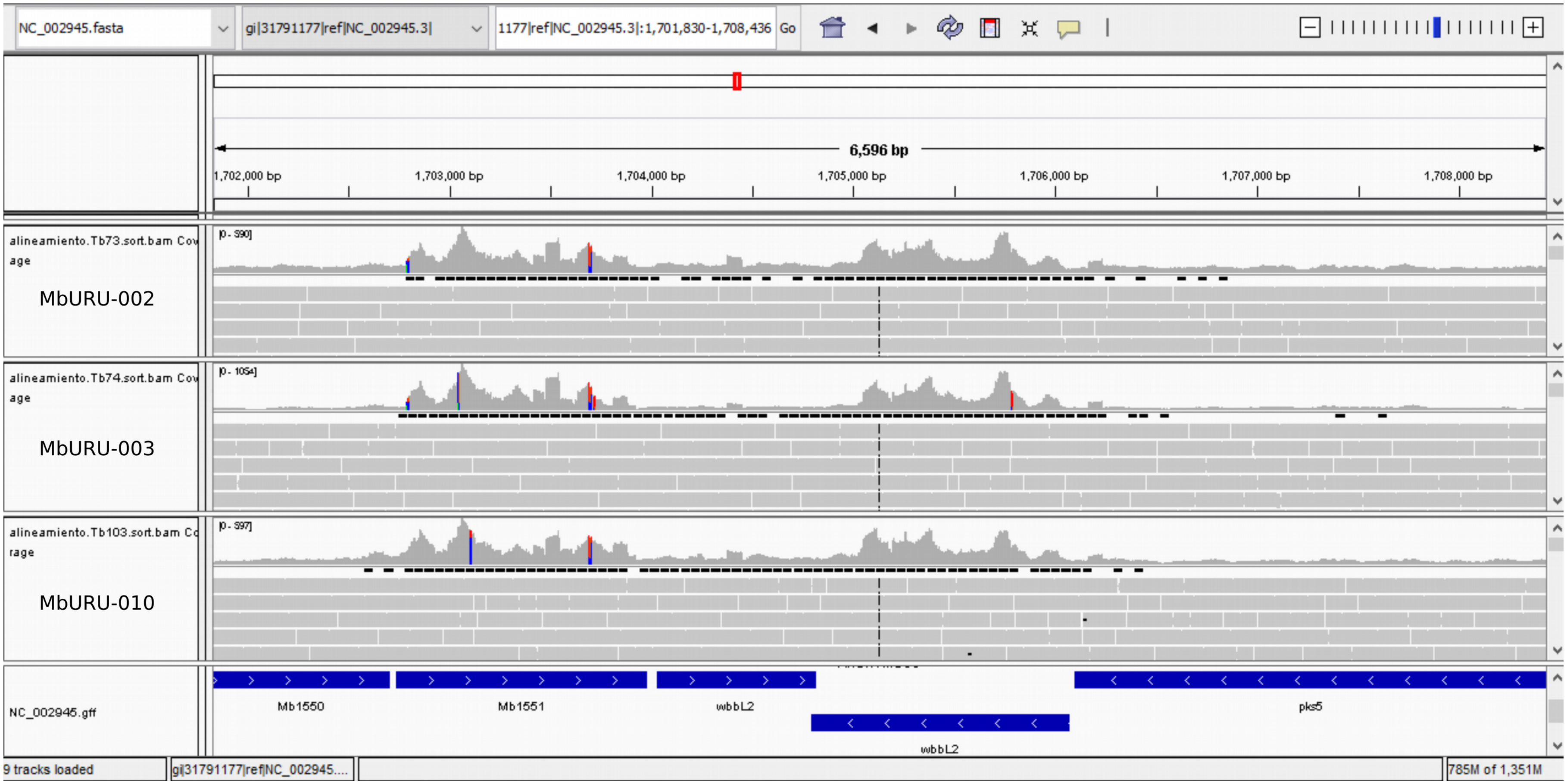

Figure S3A.- Interactive Genome Visualizer (IGV) visualization of reads aligned to reference genome AF2122/97 (exemplified by strains MbURU-002, MbURU-003 and MbURU-010), showing a region containing glycosyltransferase-coding genes Mb1551 and Mb1553c (wbbL2). Coverage is represented in the upper track of each strain as a gray bar chart, and SNPs are showed as colored bars. The average coverage for these genes can reach up to 10 times the average genome coverage.

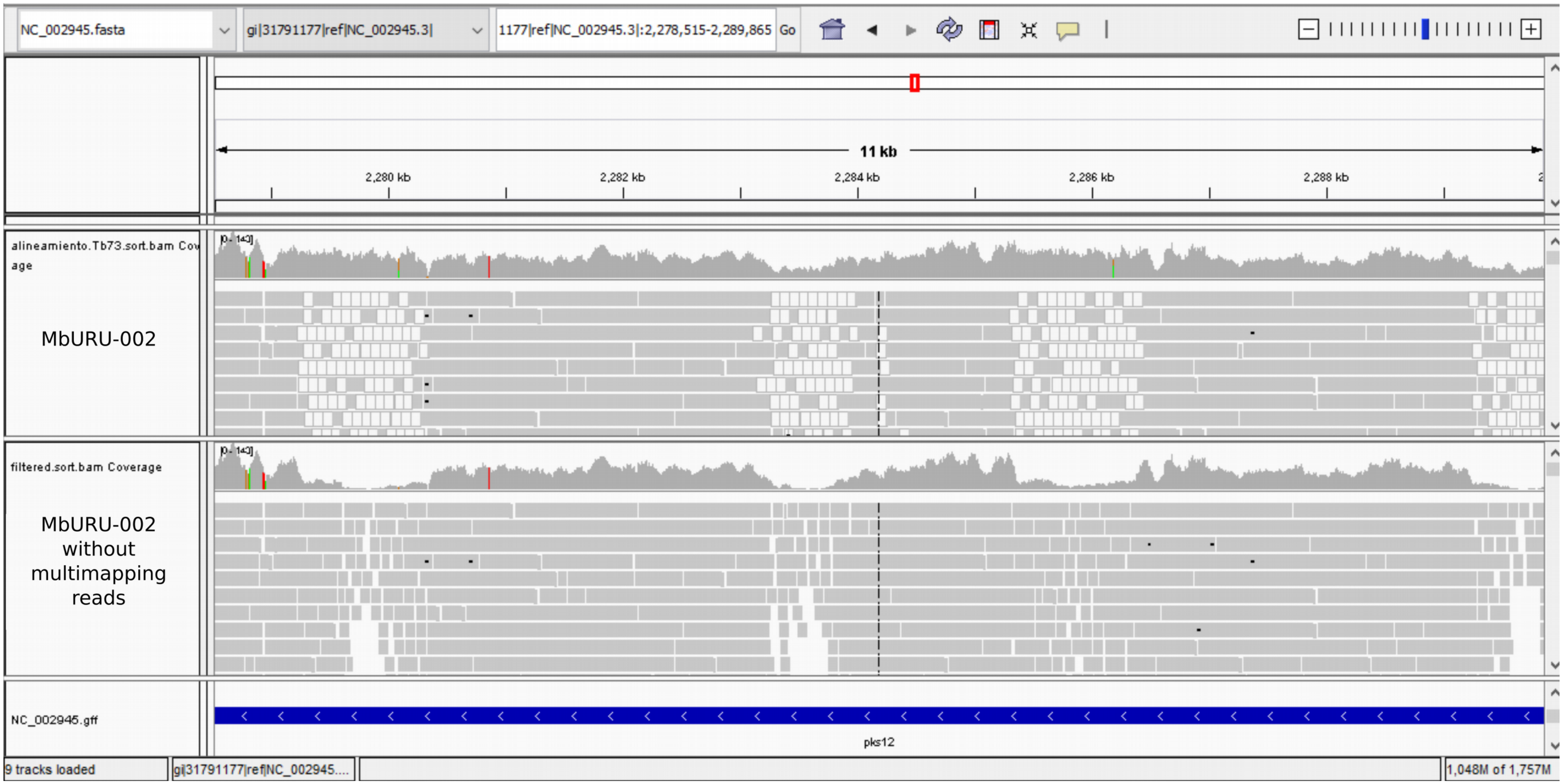

Figure S3B.- IGV visualization of reads from strain MbURU-002 aligned to reference genome AF2122/97, showing a region containing polyketide synthase *pks12* gene. The lower alignment represents only reads with mapping qualities higher than 0 and show therefore no multimapping reads, while the upper alignment represents the original data from MbURU-002. Coverage is represented in the upper track of each strain as a gray bar chart, and SNPs are showed as colored bars. Note that there are very few called SNPs in this region and some are still present once multimapping reads have been removed.
