# Supplementary material for: Whole genome sequencing of the monomorphic pathogen Mycobacterium bovis reveals local differentiation of cattle clinical isolates
Source: BMC Genomics. 2018 Jan 2;19:2. doi: 10.1186/s12864-017-4249-6 (PMC5748942; doi:10.1186/s12864-017-4249-6)
Supplement: Supplementary file 7 — Validation of in silico RD typing with PCR on 21 of the Uruguayan M. bovis strains. Figure S4b. Alignment of the sequenced reads of strain MbURU-003 against the assembled genome of the same strain. Selected pair of reads in red exemplify one of the reads that flanks both sides of a region of difference (RDbov145a) that is absent in this strain. (PDF 1643 kb) [file 12864_2017_4249_MOESM7_ESM.pdf]

RDbov130a

Strain	Amplification
MbURU-001	TRUE
**MbURU-002**	**FALSE**
MbURU-003	TRUE
MbURU-004	TRUE
MbURU-005	TRUE
MbURU-006	TRUE
**MbURU-007**	**FALSE**
MbURU-008	TRUE
MbURU-009	TRUE
**MbURU-010**	**FALSE**
**MbURU-012**	**FALSE**
MbURU-013	TRUE
MbURU-014	TRUE
MbURU-015	TRUE
MbURU-016	TRUE
**MbURU-017**	**FALSE**
**MbURU-018**	**FALSE**
MbURU-019	TRUE
MbURU-020	TRUE
MbURU-022	TRUE
MbURU-023	TRUE

RDbov145b

Strain	Amplification
MbURU-001	**FALSE**
MbURU-002	TRUE
MbURU-003	**FALSE**
MbURU-004	TRUE
MbURU-005	**FALSE**
MbURU-006	INCONCLUSIVE
MbURU-007	TRUE
MbURU-008	TRUE
MbURU-009	TRUE
MbURU-010	TRUE
MbURU-012	TRUE
MbURU-013	TRUE
MbURU-014	**FALSE**
MbURU-015	TRUE
MbURU-016	**FALSE**
MbURU-017	TRUE
MbURU-018	TRUE
MbURU-019	**FALSE**
MbURU-020	**FALSE**
MbURU-022	TRUE
MbURU-023	TRUE

Primers for RDbov130a

Forward 3’ GGCTGCTCAACGAGATGATG 5’

Reverse 3’ CATCGAACGCATCTTGAGCA 5’

Primers for RDbov145b

Forward 3’ TCAACTGGGTGGATCGTGTT 5’

Reverse 5’ GTCCTGGGAGAGATCCACAC 3’

Figure S4A.- Validation of in silico RD typing with PCR on 21 of the Uruguayan *M. bovis* strains

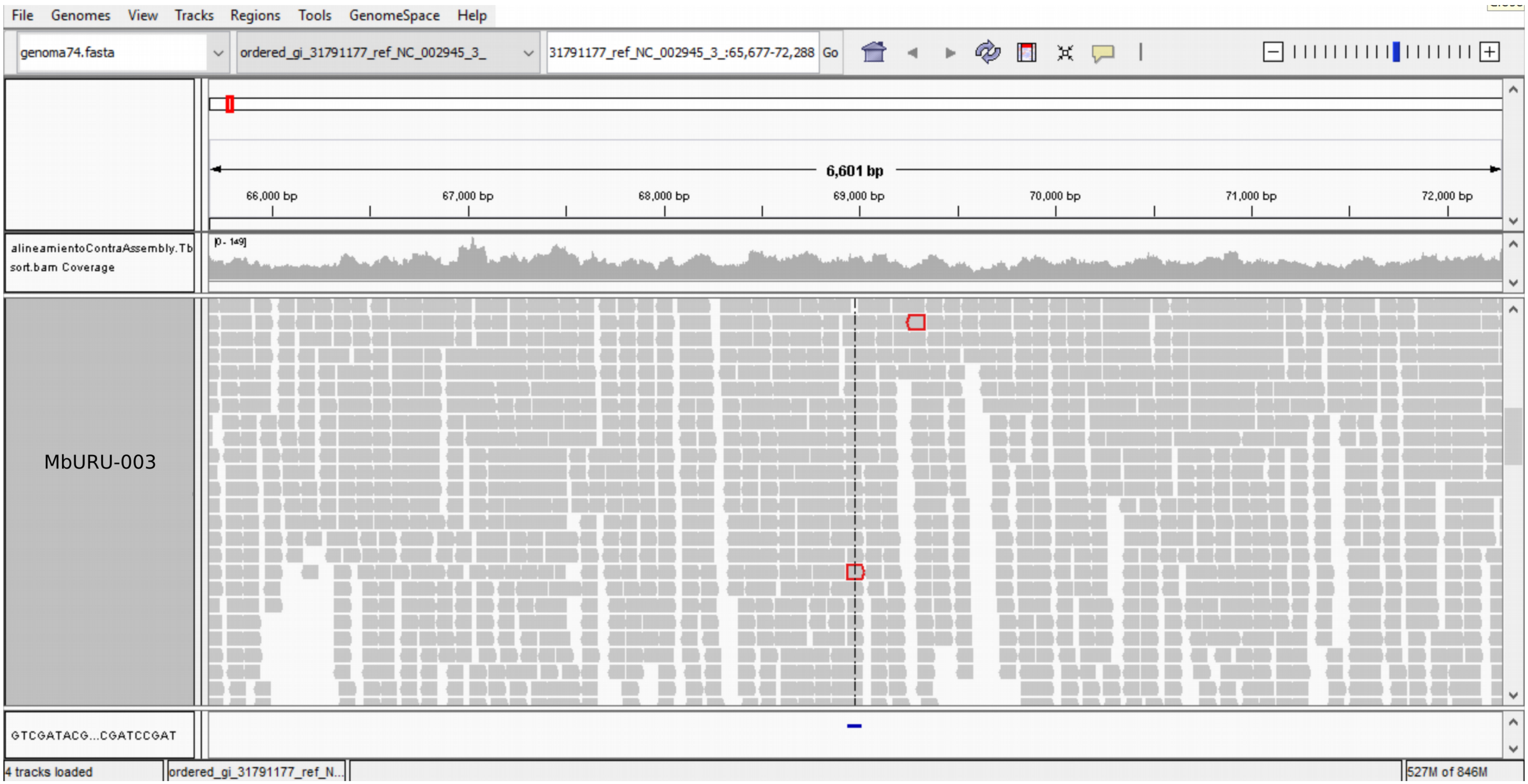

Figure S4B.- Alignment of the sequenced reads of strain MbURU-003 against the assembled genome of the same strain. Selected pair of reads in red exemplify one of the reads that flanks both sides of a region of difference (RDbov145a) that is absent in this strain.
