# Supplementary material for: Whole genome sequencing of the monomorphic pathogen Mycobacterium bovis reveals local differentiation of cattle clinical isolates
Source: BMC Genomics. 2018 Jan 2;19:2. doi: 10.1186/s12864-017-4249-6 (PMC5748942; doi:10.1186/s12864-017-4249-6)
Supplement: Supplementary file 12 — Bar plots displaying the number of genes affected per strain that are associated to the GO terms carbohydrate metabolic process and peptidoglycan-based cell wall biogenesis. (PDF 1342 kb) [file 12864_2017_4249_MOESM12_ESM.pdf]

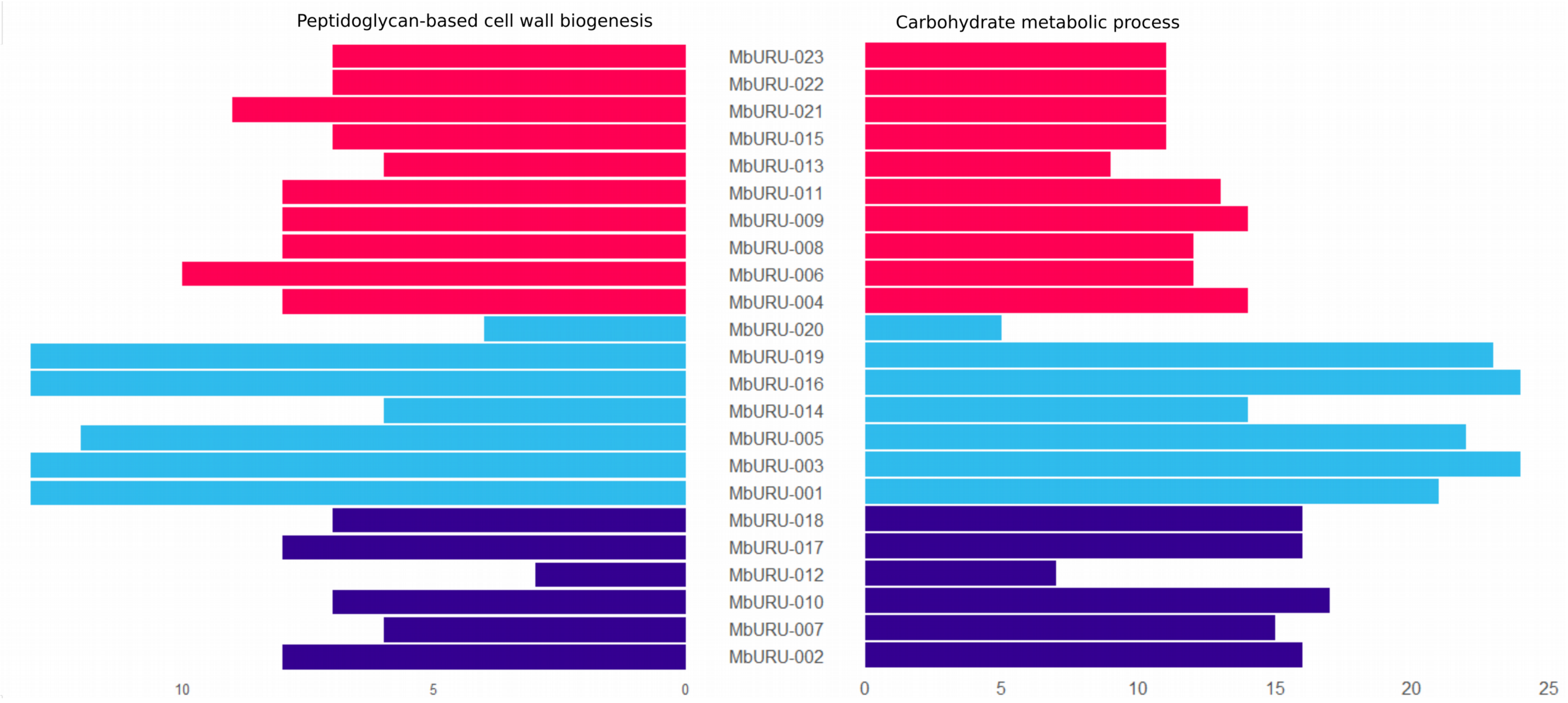

Figure S5.- Bar plots displaying the amount of genes affected per strain that are associated to the GO terms carbohydrate metabolic process and peptidoglycan-based cell wall biogenesis. Strains are grouped by URY group: URY01 (blue), URY02 (light blue) and URY03(pink). All strains were similarly responsible for the enrichment of these terms, with a light increase of genes in strains from group URY02.
